# Supplementary material for: Chromothripsis during telomere crisis is independent of NHEJ, and consistent with a replicative origin
Source: Genome Res. 2019 May;29(5):737–49. doi: 10.1101/gr.240705.118 (PMC6499312; doi:10.1101/gr.240705.118)
Supplement: Supplemental Material [file supp_gr.240705.118_Supplemental_file_1.zip › contigs/annotated_contigs/DB116/contig.2.DB116_length_301_mean_cov_4.98338870432.docx]

**DB116_length_301_mean_cov_4.98338870432**

```
ATGGTCTATTAAATATAGAGACAGCTCAGTGGGGCTGCAATATTCTCTCACAATAAACAAAGAGTGTTTGCTGCCAATAAAATATTATT
 >chrX:37920579-37920728 - E=2e-78
TATGCTTCCGTTTTGAAAACCACTCCATGAATAGCAAACAAGATACTTCCAATACTGAGA|AGTGT|ACTAGCAGATTAGTTGATGGCA
                                                                  >chrX:37919309-379194
AAATGTGTGAATACACAATCTCCTTGGATCTTGAATTGAAAAGTATACCTCAGTAATTACCTTGTATTGATATTTAGATAGATATATTT
56 - E=4e-68
TGATAGTGATATTTAGAAGTTAAAGATAAAATTCTT
```
